# 647. Optimizing SJS/TEN Management: A Five-year Retrospective Review

**DOI:** 10.1093/jbcr/irag033.484

**Published:** 2026-04-06

**Authors:** Monica Hutson, Michael Erickson

**Affiliations:** The University of Texas Medical Branch Blocker Burn Unit, Galveston, TX; The University of Texas Medical Branch Blocker Burn Unit, Galveston, TX

## Abstract

**Introduction:**

Stevens–Johnson Syndrome (SJS) and Toxic Epidermal Necrolysis (TEN) are rare but life-threatening conditions with high mortality rates and long-term complications. Management approaches differ significantly between institutions, resulting in inconsistent outcomes and limited high-quality evidence. This study offers a data-driven foundation for best practices aimed at standardizing care, optimizing resources, and enhancing survival while minimizing complications.

**Methods:**

A retrospective chart review was conducted of adults (≥18 years) admitted to the Burn Center with TEN/SJS from 2020 to 2024. Cases were first identified through the Burn Care Quality Platform (BCQP) by generating an all-cases report, filtered to non-burn etiologies categorized as rash/skin infection. Records were then sorted by the subcategory of TEN/SJS. Relevant demographics, injury details, and outcomes were retained from the report. Additional clinical data were extracted from electronic health records to evaluate trends, risk factors, and management strategies. Patients discharged within 48 hours or belonging to vulnerable populations were excluded.

**Results:**

Among the 32 patients identified, the mean age was 50.5 years, with a mean BMI of 30.6. Most patients had >10% total body surface area (TBSA) affected (75%) with mucosal involvement (81%). The average length of stay was 12 days, including 4.7 ICU days. Mortality was 9%, occurring in patients with terminal comorbidities. In each case, the culprit drug had been prescribed for the underlying condition, and death resulted from sepsis progressing to multi-organ failure. New medication exposure accounted for 78% of cases, most commonly antibiotics (34%) and anticonvulsants (22%), with Bactrim (28%) and lamotrigine (19%) as leading culprits. Common comorbidities included infection (34%), bipolar disorder (16%), and cancer (16%). Wound care was standardized to non-surgical management with an average of 4.6 wound care procedures using mild cleansers, topical antimicrobials, and silver dressings. Systemic corticosteroids were administered in 53% of cases, cyclosporine in 19%, IVIG in 9%, TNF-α inhibitors in 6%, and plasmapheresis in 3%.

**Conclusions:**

SJS/TEN in this cohort most often followed new medication exposures, particularly antibiotics and anticonvulsants, and were frequently complicated by comorbidities. Despite significant disease burden, mortality was low and confined to patients with terminal illness. Standardized non-surgical wound care proved effective, while variation in systemic therapy underscores the need for consensus in pharmacologic management.

**Applicability of Research to Practice:**

Findings support early drug identification and withdrawal, standardized wound care, and multidisciplinary burn service involvement, while highlighting the need for clearer guidance on systemic therapies.

**Funding for the study:**

N/A.

